# Comparative diversity of vascular plants in black alder floodplain and swamp forests of Central European biogeographical regions

**DOI:** 10.3897/BDJ.10.e90281

**Published:** 2022-10-26

**Authors:** Richard Hrivnák, Benjamín Jarčuška, Ivan Jarolímek, Judita Kochjarová, Jana Májeková, Katarína Hegedüšová Vantarová, Michal Slezák

**Affiliations:** 1 Institute of Botany, Plant Science and Biodiversity Center, Slovak Academy of Sciences, Bratislava, Slovakia Institute of Botany, Plant Science and Biodiversity Center, Slovak Academy of Sciences Bratislava Slovakia; 2 Institute of Forest Ecology, Slovak Academy of Sciences, Zvolen, Slovakia Institute of Forest Ecology, Slovak Academy of Sciences Zvolen Slovakia; 3 Department of Phytology, Faculty of Forestry, Technical University of Zvolen, Zvolen, Slovakia Department of Phytology, Faculty of Forestry, Technical University of Zvolen Zvolen Slovakia

**Keywords:** *Alnusglutinosa*, forest vegetation, species richness, beta and gamma diversity, Pannonian lowland, Polish Plain, Western Carpathians Mts

## Abstract

Plant species diversity of black alder-dominated forests was studied in three biogeographical regions (Alpine, Continental and Pannonian) of Central Europe. They were represented by regions of the Polish Plain (Continental), the High Western Carpathians and Matricum of the Western Carpathians (Alpine) and the Pannonian lowland (Pannonian). We analysed 35 plots per region in order to identify: i) local alpha (α) diversity defined as the counted number of plant taxa occurring in a single sampling plot, ii) amongst-site beta (β) diversity, iii) regional (γ) diversity defined as the total species richness of all sampling plots and iv) zeta diversity (ζ) as a generalisation of beta diversity. We recorded a total of 432 vascular plant taxa in all bioregions; more than 13% were alien plants. Statistically significant differences in species richness (α) of both native and alien plants were found between assemblages of the regions. The High Western Carpathians showed the highest native and the lowest alien plant species richness. Total β-diversity was high in all regions, but significantly differed amongst regions only for alien plant species. Cumulative native and alien species richness (γ) was the highest and lowest in the High Western Carpathians and Matricum of Western Carpathians, respectively. Our results identified the High Western Carpathians as a hotspot for diversity of native plants in Central European black alder-dominated forests.

## Introduction

Species diversity is an important characteristic of biotic communities related to environmental drivers and human activities (Acharya et al. 2011, Černý et al. 2013, Bonari et al. 2019). While alpha, local diversity indicates the species richness of a particular habitat, beta (the ratio between regional and local species diversity) and gamma, regional diversity (the total species diversity in a landscape) are useful tools to understand variability amongst plots and to reveal the regional richness, respectively (Whittaker 1972). In addition, zeta diversity, a recently proposed concept and metric (Hui and Mc Geoch 2014) is a generalisation of beta diversity and it represents the mean number of species common to any number of plots, where some plots correspond to a zeta order (Hui and McGeoch 2014, Riva and Mammola 2021). All of the above-mentioned diversity estimates can help to seek general diversity patterns and to better protect regional diversity and ecosystem functions (Socolar et al. 2016) in various environmental settings and spatial scales, including biogeographical regions (hereafter referred to as bioregions). Temperate broad-leaved forests showed regional differences in species richness and composition of vascular plants due to biogeography and climatic contrasts (Douda et al. 2016, Večeřa et al. 2019). The bioregions thus provide ideal opportunities for comparative analyses of plant diversity. Central Europe contains three important European bioregions, namely Alpine and Continental which capture large area of the continent and the Pannonian bioregion which is specific for Central and southern Europe (Cervellini et al. 2020).

Research on plant diversity in forests has a long tradition in Central Europe, especially using vegetation-plot data in the most abundant beech forests (e.g. Sabatini et al. 2018). Wetland forests have also been a subject of biodiversity analyses, which have been considered mainly an effect of site heterogeneity, environmental history and human impact (Slezák et al. 2017, Douda et al. 2018, Pielech 2021). Comparisons of species diversity between habitats and/or regions have been studied mainly in grasslands or various freshwater habitats (e.g. Sekulová et al. 2012, Janišová et al. 2014, Bubíková and Hrivnák 2018), but this approach is relatively rare for forest vegetation (e.g. Ujházyová et al. 2016, Večeřa et al. 2019).

Floodplain and swamp forests dominated by black alder (*Alnusglutinosa*) are widespread throughout Europe (Douda et al. 2016, Chytrý et al. 2020, Preislerová et al. 2022). They represent natural vegetation at many sites in river floodplains and are reported amongst the important habitats for nature conservation objectives (European Habitats Directive 92/43/EEC). In spite of their important ecological functions and ecosystem services, they are still under strong negative pressure from human activities and are susceptible to plant invasions (Richardson et al. 2007, Slezák et al. 2022). They provide a suitable opportunity for a biodiversity study of native and alien species diversity across a broad geographical scale due to their relatively common distribution in Central Europe and widespread alien plants. In accordance with these circumstances, our study aimed: i) to determine diversity patterns of native and alien vascular plants in floodplain and swamp forests, ii) to compare α, β, γ and ζ diversity amongst bioregions of Central Europe and iii) to identify the plant diversity hotspot for these habitats in the studied region. Niche diversification hypothesis states that total diversity of a community is a function of the total range of habitat conditions (Roberts and Gilliam 1995). Some plants are not highly specialised and all require the same resources; thus they subdivide their niche by variation in resources, elevation, slope and soil type. In accordance with this theory, we assume that high altitudinal, climatic and geological heterogeneity would have positive effect on plant diversity. Therefore, the northern part of the Western Carpathians Mts (WeCa) in the Alpine bioregion with the highest environmental heterogeneity would have the highest richness of native species and the lowest diversity of alien species, compared to other three regions. On the contrary, alien diversity would increase at lower elevational bioregions following previously recognised trends that the number of alien plant species decreases with increasing elevation mainly due to less intensive human activities, the resistance of mountain ecosystems to the spreading of non-native plants and, generally, ecological demands of alien plants (e.g. Pauchard et al. 2009, Medvecká et al. 2014, Sabatini et al. 2018, Slezák et al. 2020a, Di Musciano et al. 2021).

## Methods

### Study site

**Figure 1.**
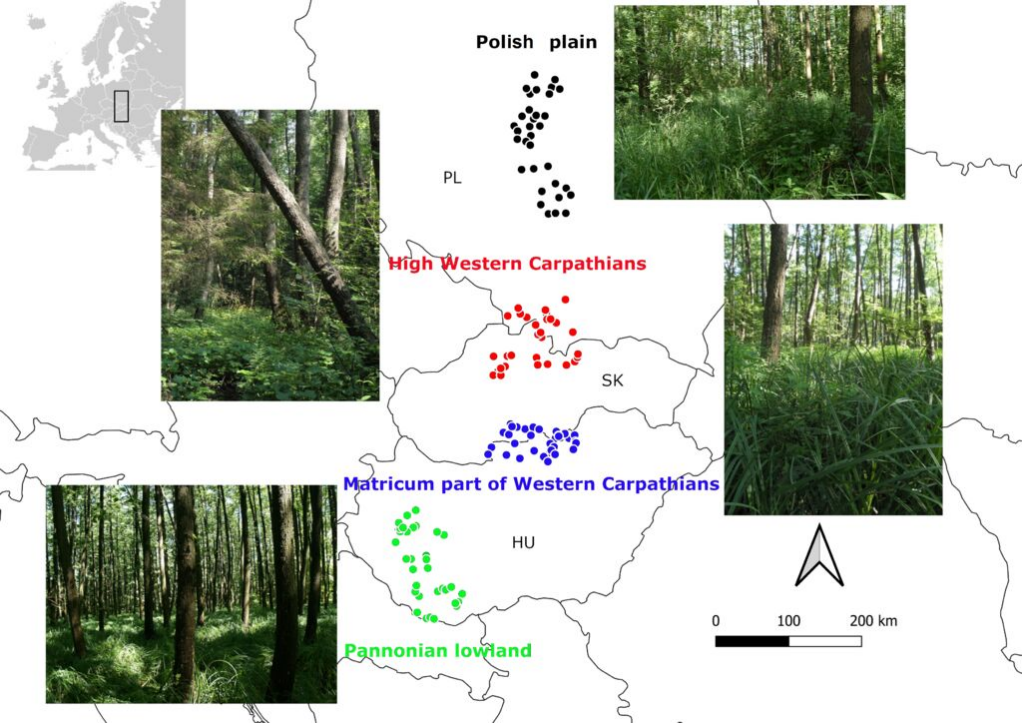
Map of the studied regions with dots indicating sampled vegetation plots.

Our study area is situated along a latitudinal gradient (45.8085° to 52.6115°) in Central Europe (from southern Hungary and Slovakia to central Poland), including the Alpine, Continental and Pannonian bioregions (Cervellini et al. 2020). We split the Alpine region into two separate units due to high elevational variability at a small spatial scale, steep climatic (elevational) and ecological gradients. For this purpose, the study area consists of four analysed regions, i.e. the Pannonian lowland in the Pannonian bioregion (Pannonia), the Matricum as part of the Western Carpathians (MatrWeCa) in the foothills of the Pannonian/Alpine bioregion, the High Western Carpathians (HighWeCa) in the Alpine bioregion and the Polish Plain (PolaPai) in the Continental bioregion (Fig. 1). The Pannonian lowland includes the lowland and hilly region of central and southern Hungary with mountains such as Bakony and Mecsek Mts (45.8085° to 47.1271°). The Matricum in the Western Carpathians consists of southern Slovakia and northern Hungarian basins and mountains such as Cerová vrchovina, Cserhát, Mátra and Bükk Mts (47.8565° to 48.2861°). The High Western Carpathians form the Inner Carpathian basins in Slovakia and high mountain ranges such as Nízke and Vysoké Tatry Mts, Veľká and Malá Fatra Mts and Beskyd/Beski's Mts in Slovakia and Poland (48.8737° to 49.7251°). The Polish Plain includes large lowland and low mountains, for example, Wisoczyzna Bechlatowska and Klodawska (50.9123° to 52.6115°). The regions are arranged along the climatic gradient from the warmest and driest Pannonian, through MatrWeCa and PolaPlai to HighWeCa with the highest and lowest values of precipitation and temperature, respectively (Table 1). Elevation increases from the Polish Plain and the Pannonian lowland through the Matricum of WeCa to high-elevational areas of the HighWeCa (Table 1). The highest geological bedrock heterogeneity was found in HighWeCa, followed by MatrWeCa, PolaPlai and Pannonian (https://macrostrat.org/map/#/z=5.1/x=21.9117/y=50.6320/bedrock/lines/).

The study was conducted in floodplain forests (*Alnionincanae* Pawłowski et al. 1928) and alder swamp forests (*Alnionglutinosae* Malcuit 1929) dominated by *Alnusglutinosa*, which cover azonal/intrazonal forest vegetation of periodically flooded floodplain areas and waterlogged sites on the banks of lentic ecosystems, marsh and mire margins (Douda et al. 2016). Generally, the alliance *Alnionincanae* comprises the ash-alder stream and seepage forests dominated by *Alnusglutinosa* and *Fraxinusexcelsior* in the nemoral and hemiboreal zones. They are distributed from oceanic Western Europe to continental Eastern Europe within a broad range of continentality and elevation (Douda et al. 2016). The alliance *Alnionglutinosae* includes vegetation of swamps mostly dominated by tree species *Alnusglutinosa*. The herb layer of these forests is composed of tall sedges and wetland herbs broadly distributed in the nemoral and hemiboreal zones of Europe and with rare occurrence in the Mediterranean Basin, as well (Douda et al. 2016, Mandžukovski et al. 2021).

### Field sampling

Vegetation data were sampled in physiognomically and structurally homogeneous mature forest stands with a dominant cover (i.e. canopy cover of more than 50%) of *Alnusglutinosa* in the tree layer. Sampling plots were selected in the field based on literature sources (published data on the distribution of vegetation types) and the author’s expertise. All plots had uniform size (400 m^2^) with square or rectangular shapes driven mainly by river valleys’ morphology. Sampling density followed the environmental heterogeneity and the presence of different local vegetation types (Hrivnák et al. 2015, Slezák et al. 2017). We collected 173 plots using the traditional European phytosociological approach (Dengler et al. 2008) in vegetation seasons (from June to August) of 2010–2019. For each plot, vascular plants were recorded with their cover in three layers (tree, shrub and herb) using a modified Braun-Blanquet sampling scale (Barkman et al. 1964, Van Der Maarel 1975). The geographical position and elevation of the plot centre were measured by the GPS receiver.

### Data processing and analysis

Each plot was assigned to a biogeographical region defined by the European Environmental Agency (Cervellini et al. 2020). Vegetation data from all sampling plots were stored in Turboveg database software (Hennekens and Schaminée 2001) and then exported to Juice software (Tichý 2002). To harmonise the dataset, we randomly selected 35 plots per region and unified species nomenclature according to the Euro+Med PlantBase (https://www.emplantbase.org/home.html). The same species presented in different layers were merged into one species record. Vascular plants not determined at the species level were deleted (25 taxa in genus level with 57 occurrences). The following plant taxa were merged into aggregates or a broadly defined level: *Galeobdolonluteum* agg. (*G.luteum*, *G.montanum*), *Myosotispalustris* agg. (*M.laxiflora*, *M.nemorosa*), *Pulmonariaofficinalis* agg. (*P.officinalis*, *P.obscura*), *Pyruscommunis* agg. (*P.communis*, *P.pyraster*), *Solidagocanadensis* agg. (*S.canadensis*, *S.gigantea*) and *Stellariamedia* agg. (*S.media*, *S.neglecta*). Plant species recorded in 140 plots were classified as native and alien plants. Alien taxa (archaeophytes and neophytes) were identified according to Medvecká et al. (2012) for the Alpine bioregion, Zając and Zając (2011), Tokarska-Guzik et al. (2012), Tokarska-Guzik et al. (2014)for the Continental bioregion and Terpó et al. (1999), Balogh et al. (2004), Botta-Dukát and Balogh (2008) and Bartha (2020) for the Pannonian bioregion. Only presence-absence (i.e. incidence) data for vascular plant species were used in the next analyses.

We used two climatic variables (mean annual temperature and mean annual precipitation) as characteristics for the bioregions. They were retrieved from WorldClim version 2 (Fick and Hijmans 2017; http://www.worldclim.org) on a grid background with an accuracy of spatial resolution in 30 seconds (ca. 0.55 km^2^). These variables are derived from monthly temperature and rainfall values over a long-term period (1970-2000).

Local alpha (α) diversity was defined as the counted number of plant taxa in a single sampling plot. Differences in local species richness between regions were tested using the Kruskal-Wallis (rank sum) test. Pairwise post-hoc comparison of differences between regions was conducted using the Wilcoxon (rank sum) test with Holm correction for the use of multiple analyses.

We assessed the effect of regional identity on the amongst-site beta (β) diversity of plant species between assemblages using a multivariate test for homogeneity of group dispersions implemented in the “betadisper” function in the vegan package (version 2.5-5; Oksanen et al. 2019). The Jaccard dissimilarity (binary) index was used as the input distance structure (Schroeder and Jenkins 2018). ANOVA (followed by a permutation-based test, the permutest function from the package vegan) was performed to test differences in beta diversity amongst the bioregions by comparing distances from individual sampling plots to their bioregion centroid (Anderson et al. 2006). Next, we decomposed dissimilarity coefficients (Jaccard binary index based multiple-plot dissimilarity) using Podani family decomposition (Podani and Schmera 2011, Legendre 2014) into species replacement and species richness difference components for each of the four regions. Nestedness was calculated as the relativised nestedness index (Podani and Schmera 2011). We used the beta.div.comp function from the R package adespatial (version 0.3-14; Dray et al. 2021). For alien plant species, plots without alien species were excluded from analyses.

Regional (γ) diversity corresponded to the total species richness of all sampling plots within a given sub-region. We used a unified framework linking rarefaction (interpolation) and prediction (extrapolation) of Hill numbers (Hill 1973) with species accumulation curves (Chao et al. 2014, Hsieh et al. 2016). We estimated Hill numbers for sample-based incidence data, which are diversity indices that consider species richness and species relative incidence in an assemblage. To assess the statistical significance of differences in diversity indices amongst regions, a bootstrapping method (200 bootstraps) was applied to generate 95% confidence intervals (MacGregor-Fors and Payton 2013) with the iNEXT function (the R package iNEXT, version 2.0.20; Hsieh et al. 2020). Extrapolation is only robust to twice the minimum sample size (i.e. 35 plots – sampling units) for species richness, i.e. we calculated accumulation curves for 70 sampling units. Sampling completeness was assessed using the function estimated in the iNEXT package.

Zeta diversity (ζ) is a generalisation of beta diversity and it was determined as follows: i) zeta1 is the average number of species per plot (i.e. species richness or alpha diversity), ii) zeta2 is the average number of species shared by any two plots (i.e. the reverse of species turnover or beta diversity), iii) zeta3 is the average number of species shared by any three plots, etc. The number of shared species across plots is negatively associated with the order of zeta – zeta decline. The zeta ratio – retention rate (ratio of the number of species shared by i-plots to the number of species shared by i - 1-plots; McGeoch et al. 2017) quantifies the relative turnover rate of rare and common species and, thus, may allow us to distinguish apparently similar zeta declines or to compare zeta declines with different richness values. The Zeta.decline.ex function from the R package zetadiv (version 1.2.0; Latombe et al. 2018, Latombe et al. 2020) was used to calculate the zeta decline and zeta ratio for native and alien species from the four regions. For alien plant species, plots without aliens were excluded from analyses.

All analyses were performed using R software version 3.6.3 for Windows (R Development Core Team 2020) implemented in the RStudio 1.2.1335 environment (RStudio Team 2019) using presence-absence (incidence) data on species occurrence within plots. Statistical significance is considered at P < 0.05.

## Results

### Alpha diversity

We recorded a total of 432 vascular plant taxa in all bioregions; more than 13% were alien plants. Except for *Alnusglutinosa*, the most frequent plants were exclusively native species, such as *Urticadioica* (86%), *Galiumaparine* (69%), *Sambucusnigra* (56%), *Geumurbanum* (54%), *Rubuscaesius* (54%) and *Poatrivialis* (50%). The most common aliens were neophytes *Impatiensparviflora* (25%), *Ribesrubrum* agg. (24%), *Bidensfrondosa* (15%) and archaeophyte species *Chelidoniummajus* (19%). All regions had 238 taxa in common, with 37, 69, 35 and 31 taxa occurring only in PolaPlai, HighWeCa, MatrWeCa and Pannonia regions, respectively.

**Figure 2.**
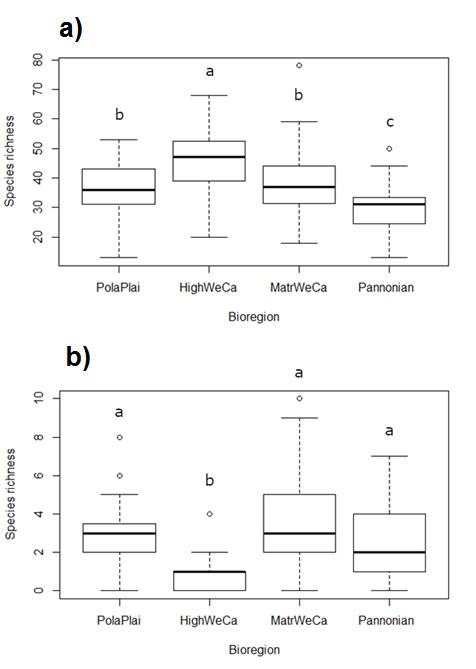
Native (a) and alien (b) plant species richness (alpha diversity) for each region (PolaPlai – Polish Plain, HighWeCa – High Western Carpathians, MatrWeCa – Matricum of Western Carpathians, Pannonian – Pannonian lowland). Different letters indicate statistically significant differences at P < 0.05.

Statistically significant differences in local species richness (i.e. alpha diversity) of both native and alien plants were found between assemblages of the four regions (χ^2^ = 36.591, P < 0.001 and χ^2^ = 39.041, P< 0.001, respectively). Native species richness was significantly lower in the Pannonian region than in the other three regions. Species richness in PolaPlai was significantly lower compared to HighWeCa, but higher than in the Pannonian region. The HighWeCa region showed higher species richness than the other three regions. Contrary to the pattern of species richness observed for native species, alien species richness was significantly lowest in the HighWeCa region (0.7 per plot on average), while the other regions did not differ significantly (2.4 PolaPlai, 3.4 MatrWeCa and 2.1 Pannonian; Fig. 2).

### Beta diversity

**Figure 3.**
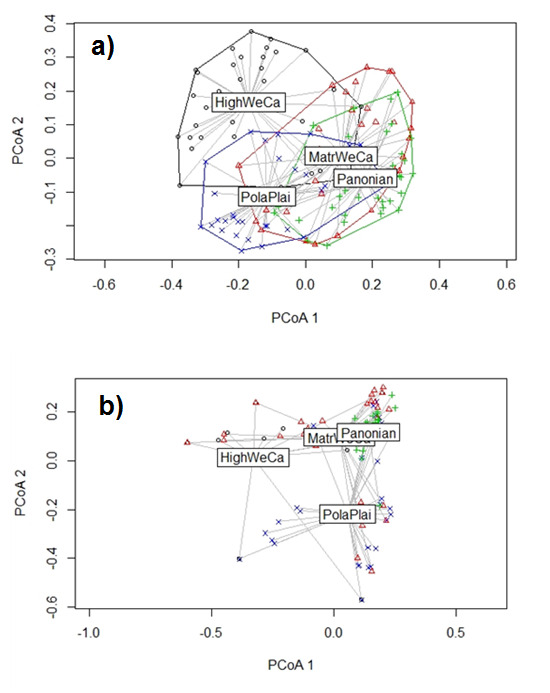
Beta diversity of native (a) and alien (b) plant species assemblages in the bioregions by running the betadisper function. ANOVA was applied to test how these distances differed amongst the communities. PCoA1 and PCoA2 are the first and second sort axes in the “betadisper” analysis, respectively. PolaPlai – Polish Plain, HighWeCa – High Western Carpathians, MatrWeCa – Matricum of Western Carpathians, Pannonian – Pannonian lowland.

Betadisper analysis followed by ANOVA showed no significant differences in beta diversity of the native plant assemblages in the four regions (F = 0.1596, P = 0.929) (Fig. 3a). Total β-diversity was high in all regions (Table 2). The results of decomposing total dissimilarity into replacement and richness difference components showed that dissimilarity of native plant assemblages was determined mainly by species replacement rather than species richness difference across plots within the four regions, reflecting the continuous turnover of native plant species in the regions and suggesting environmental filtering. Species replacement comprised more than 70% of multi-plots dissimilarity in each of the four regions (Table 2).

In the case of alien species, betadisper analysis showed significant differences in alien plant assemblages in studied regions (F = 6.4943, P < 0.001; Fig. 3b). The results of total dissimilarity decomposition into replacement and richness difference components indicated that richness difference were relatively more important component of dissimilarity for alien plants in assemblages than for native species (Table 2). Nestedness of alien species was larger in regions with higher species richness of aliens (Table 2, Fig. 3b).

### Gamma and Zeta diversity

**Figure 4.**
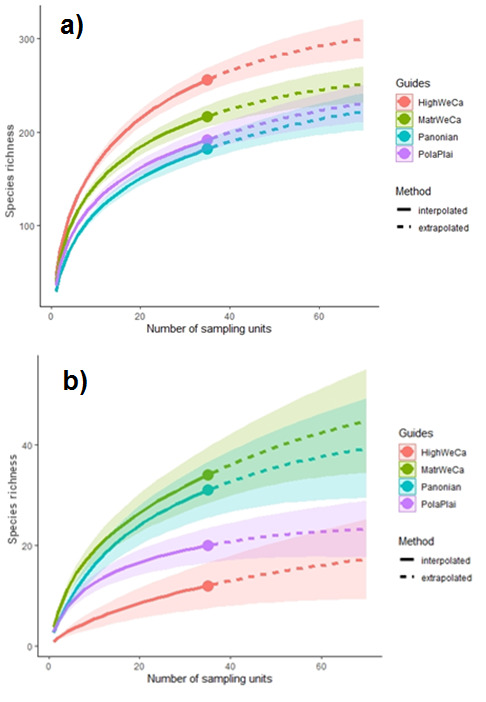
Sample-size-based rarefaction (solid line segment) and extrapolation (dashed line segment) curves for species richness (Hill number q = 0) with 95% confidence intervals of native (a) and alien (b) vascular plant species of the four studied regions. PolaPlai – Polish Plain, HighWeCa – High Western Carpathians, MatrWeCa – Matricum of Western Carpathians, Pannonian – Pannonian lowland.

Native species richness was highest in the HighWeCa, followed by MatrWeCa and PolaPlain regions. The lowest values were found in the Pannonian region (Fig. 4a). In contrast, species accumulation curves for alien plants showed the lowest values in the HighWeCa and PolaPlain regions. These values significantly differed from those found in the MatrWeCa and Pannonian regions (Fig. 4b).

**Figure 5.**
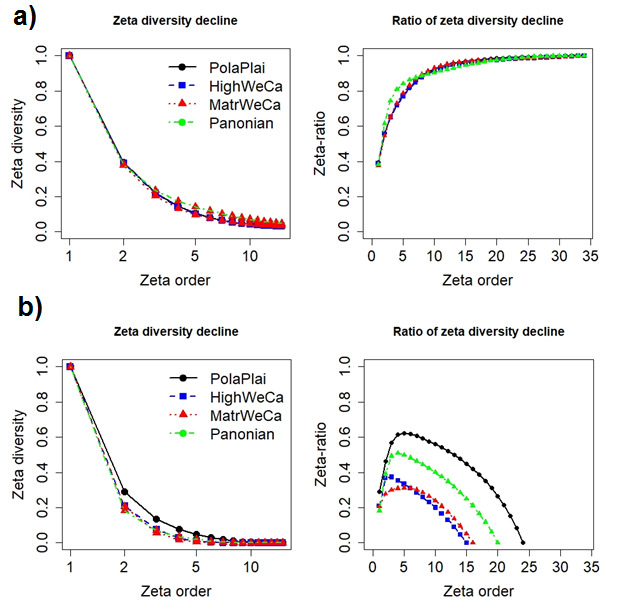
Zeta diversity of native (a) and alien (b) plant species. Zeta decline (rescaled between 0 and 1 for comparison; the x-axis is on a log-scale and stops at order 15 to highlight differences between assemblages of the four regions) (left) and the corresponding zeta ratio computing the retention rate (right). The x-axis of the zeta decline is on a log-scale for clarity. PolaPlai – Polish Plain, HighWeCa – High Western Carpathians, MatrWeCa – Matricum of Western Carpathians, Pannonian – Pannonian lowland.

The shift in plant assemblage structure of the four regions, estimated by the shape of the zeta declines, is similar for the native species because the zeta declines have the same pattern. The same is true for the shape of the zeta ratio between regions (Fig. 5a). The pattern of association between zeta ratio and zeta order for native plant species (asymptotic curve of zeta ratio) suggests that there is a group of native species common for all sites within the four regions. However, the decrease of zeta diversity of alien plant species is sharper compared to native plant species for MatrWeCa and Pannonian regions. Both regions also showed more pronounced differences. Alien species from the MatrWeCa region had lower retention rate compared to the other three regions. HighWeCa is a mountainous region with the lowest species number of alien species, which is reflected in a high retention rate but the greatest decline in zeta ratio (Fig. 5).

## Discussion

Our results suggest that the highest alpha and gamma diversity of native vascular plants was found in the HighWeCa region. There are at least three mutually non-exclusive reasons which could explain these patterns. First, environmental heterogeneity (i.e. high climatic and bedrock variability in our study) should have a positive effect on plant diversity. We assume, in accordance with niche diversification hypothesis (Roberts and Gilliam 1995), the highest variability in elevation and related climatic features (Table 1) accompanied by variation in geological bedrock observed in HighWeCa (see also Ujházyová et al. 2016) facilitates plant species diversity. Second, the spread of typical plants of adjacent habitats, that are well-adapted to high precipitation and low air temperatures, increases species diversity of alder-dominated wetland forests in HighWeCa. Moisture-demanding species of surrounding habitats, such as fir-beech and fir-beech-spruce forests, (sub)mountain wetlands and grasslands in HighWeCa, are also well adapted to the ecological conditions of floodplain forests (Valachovič et al. 2021). They enter the understorey of these forests relatively easily. These plants are generally present in the species composition of mountain riparian and swamp alder forests in the northern part of the Western Carpathians (e.g. Slezák et al. 2020b). Therefore, they enrich the diversity of studied forests. Third, swamp forests are relatively rarer in the northern part of the Western Carpathians than in the southern regions of the study area (Douda et al. 2016). It has been previously found that black alder swamp forests are species poorer in vascular plants than riparian forests of particular regions (e.g. Slezák et al. 2020b, Mandžukovski et al. 2021). All the above statements predestine the HighWeCa region as a hotspot of native species richness in the study area.

We also provided evidence that the MatrWeCa bioregion showed the highest alpha diversity of alien plants. In accordance with outcomes of previous biodiversity studies (e.g. Becker et al. 2005, Medvecká et al. 2014), we hypothesised that low-altitudinal regions (PolaPlai and Pannonian) would be richer in alien species than the other two regions of the Western Carpathians. However, our results did not confirm the highest alien plant richness in the bioregions with the lowest elevation. Two regions with lower mean values of elevation (i.e. PolaPlai and Pannonian) had lower alien plant richness than MatrWeCa (see Fig. 2), but the differences were not statistically significant. In addition to altitude and analysed climatic variables, we suppose that other predictors, such as landscape utilisation, human population density and long-term agriculture use of the MatrWeCa region, play an important role for alien richness pattern. Relatively high air temperature and low precipitation (https://sites.ualberta.ca/~ahamann/data/climateeu.html), as well as a high proportion of agricultural and urban areas in Slovakia are typical for this region. These factors are suitable for the survival of alien plant species. The response of alien richness to elevation is driven by more adverse climatic conditions than in other studied bioregions and decreasing anthropogenic disturbance and propagule pressure towards higher elevational areas (Zhang et al. 2015). The anthropogenic effect on non-native species diversity along the elevational gradient may be amplified by the role of soil type, relief and landscape structure (Szymura et al. 2018). The relationship between alien diversity and elevation thus reflects the synergic influence of multiple environmental factors that change with increasing elevation.

The overall β-diversity determined by the Jaccard dissimilarity index showed similar high values for native and alien plants in all studied regions. However, the heterogeneity of plant assemblages’ dissimilarity amongst regions was significant only for the alien species. Only 1.2% of all recorded species were found in more than 50% of plots and almost 75% of plants were found in less than 10% of plots. Moreover, more than a quarter (26.6%) of all plant species were recorded in only one plot and this high ratio of more or less random species is important. Species composition mirrors local ecological conditions, but regional factors, such as climatic characteristics or expansion patterns of *Alnusglutinosa*, also contribute to β-diversity (Douda et al. 2018, Hrivnák et al. 2020). For example, the time of the post-glacial expansion of dominant *Alnus* trees was the most important regional variable influencing the components of local β-diversity (Douda et al. 2018). The high total (gamma) species diversity of floodplain and swamp forests dominated by *Alnusglutinosa* and the average species richness per plot are typical features of these habitats due to the surface micro-topographical (hydrological) heterogeneity, typical mainly for swamp forests (Douda et al. 2012, Pielech 2015, Slezák et al. 2017, Slezák et al. 2020b).

## Conclusion

The High Western Carpathians were found as a hotspot for diversity of native vascular plants in Central European riparian and swamp alder forests, while the other three studied regions (the Polish Plain, the Matricum of Western Carpathians and the Pannonian lowland) hosted much fewer species. On the contrary, alien plants were more abundant in the remaining three regions with the highest number being found in the Matricum of Western Carpathians.

## Acknowledgements

The research was supported by the Science Grant Agency of the Ministry of Education of the Slovak Republic and the Slovak Academy of Sciences (VEGA 2/0016/19).

## Figures and Tables

**Table 1.** Descriptive statistics of altitudinal and climatic characteristics for sampled vegetation plots in the riparian and swamp alder forests in studied biogeographical regions. The climatic variables were retrieved from WorldClim version 2 (Fick and Hijmans 2017; http://www.worldclim.org).

	**Mean**	**SD**	**Min**	**Max**
**Altitude [m**]				
Pannonian lowland	149.0	37.3	97.6	246.9
Matricum Western Carpathians	249.3	65.0	112.4	384.3
High Western Carpathians	561.5	139.7	294.0	869.6
Polish Plain	143.6	48.6	62.0	224.6
**Mean annual temperature [°C**]				
Pannonian lowland	10.6	0.3	10.0	11.2
Matricum WeCa	8.9	0.5	8.0	10.6
High WeCa	6.6	0.8	4.9	8.3
Polish Plain	8.2	0.2	7.9	8.5
**Total annual precipitation [mm**]				
Pannonian lowland	622.3	33.9	563.0	702.0
Matricum WeCa	558.1	37.2	518.0	669.0
High WeCa	850.1	74.0	679.0	1025.0
Polish Plain	537.6	20.8	514.0	583.0

**Table 2.** Jaccard dissimilarity (total β -diversity), its species replacement and species richness differences fractions (Podani family of coefficients) and relativised nestedness index for presence-absence data of native and alien plants, based on each region.

**Bioregion**	**Total β-diversity**	**Replacement**	**Richness differences**	**Nestedness**
**Native plant species**				
Polish Plain	0.38	0.73	0.27	0.60
HighWeCa	0.38	0.76	0.24	0.56
MatrWeCa	0.38	0.72	0.28	0.58
Pannonian lowland	0.38	0.75	0.25	0.56
**Alien plant species**				
Polish Plain	0.39	0.58	0.42	0.60
HighWeCa	0.34	0.63	0.37	0.71
MatrWeCa	0.43	0.55	0.45	0.45
Pannonian lowland	0.44	0.62	0.39	0.32
